# Protective immunity induced by peptides of AMA1, RON2 and RON4 containing T-and B-cell epitopes via an intranasal route against toxoplasmosis in mice

**DOI:** 10.1186/s13071-015-0636-5

**Published:** 2015-01-13

**Authors:** Tie-E Zhang, Li-Tian Yin, Run-Hua Li, Hai-Long Wang, Xiao-Li Meng, Guo-Rong Yin

**Affiliations:** Research Institute of Medical Parasitology, Shanxi Medical University, Xinjian South Road, Taiyuan, Shanxi Province 030001 China; Department of Clinical Laboratory, Central Hospital of the 12th Bureau Group of China Railway, Taiyuan, Shanxi 030053 China; Department of physiology, Key Laboratory of Cellular Physiology Co-constructed by Province and Ministry of Education, Shanxi Medical University, Taiyuan, Shanxi 030001 China; Department of Biology, Taiyuan Normal University, Taiyuan, Shanxi 030031 China

**Keywords:** *Toxoplasma gondii*, AMA1, RON2, RON4, Peptide epitope, Mucosal vaccine

## Abstract

**Background:**

*Toxoplasma gondii* is a ubiquitous protozoan intracellular parasite, the causative agent of toxoplasmosis, and a worldwide zoonosis. Apical membrane antigen-1 (AMA1) and rhoptry neck protein (RON2, RON4) are involved in the invasion of *T. gondii*.

**Methods:**

This study chemically synthesized peptides of TgAMA1, TgRON2 and TgRON4 that contained the T- and B-cell epitopes predicted by bioinformatics analysis. We evaluated the systemic response by proliferation, cytokine and antibody measurements as well as the mucosal response by examining the levels of antigen-specific secretory IgA (SIgA) in the nasal, vesical and intestinal washes obtained from mice after nasal immunization with single (AMA1, RON2, RON4) or mixtures of peptides (A1 + R2, A1 + R4, R2 + R4, A1 + R2 + R4). We also assessed the parasite burdens in the liver and brain as well as the survival of mice challenged with a virulent strain.

**Results:**

The results showed that the mice immunized with single or mixed peptides produced effective mucosal and systemic immune responses with a high level of specific antibody responses, a strong lymphoproliferative response and significant levels of gamma interferon (IFN-γ), interleukin-2 (IL-2) and IL-4 production. These mice also elicited partial protection against acute and chronic *T. gondii* infection. Moreover, our study indicated that mixtures of peptides, especially the A1 + R2 mixture, were more powerful and efficient than any other single peptides.

**Conclusions:**

These results demonstrated that intranasal immunisation with peptides of AMA1, RON2 and RON4 containing T- and B-cell epitopes can partly protect mice against toxoplasmosis, and a combination of peptides as a mucosal vaccine strategy is essential for future *Toxoplasma* vaccine development.

## Background

*Toxoplasma gondii* is a significant obligate intracellular protozoan parasite because it infects a wide variety of warm-blooded animals, including humans [1]. Infection in humans can cause severe ocular, neurologic and sometimes systemic disease, especially in immunocompromised and congenitally infected individuals [2-4]. *Toxoplasma gondii* can sometimes be acquired congenitally by a newborn from an infected mother, congenital toxoplasmosis may cause abortion, neonatal death or fetal abnormalities [4,5]. Currently, *T. gondii* control depends primarily on chemotherapy, but the available medicines have many severe side effects. The development of safe and effective vaccines is the best strategy to prevent toxoplasmosis [6,7].

Successful invasion is the first and important step for the parasite to infect the host cells. The invasion process by *T. gondii* involves a moving junction (MJ) formed between the apex of the parasite and the host cell membrane [4], and the MJ structure is used by *T. gondii* to propel itself inside the cell using a gliding motion. Apical membrane antigen-1 (AMA1) plays a central role during invasion of *T. gondii* [8-11] as a member of the moving junction complex [12,13]. Rhoptry neck protein 2 (RON2) is present not only at the nascent MJ [13] but also at the progressing MJ, where it co-localizes with other RON partners. Therefore, RON2 is present at the host cell-parasite interface during the complete invasion process [14]. AMA1 uses the RON2 as a receptor, and the AMA1-RON2 interaction is a key for invasion [14,15]. Moreover RON4 participates in MJ formation and is an indispensable component of the MJ complex [12,13,16].

Because MJ components play an essential role in invasion by *T. gondii*, the proteins in the MJ structure may be potential vaccine candidates. AMA1 has been a key malaria vaccine candidate and induces antibodies that inhibit invasion and confer protection in animals [11,14]. In addition, a plasmid that encodes *T. gondii* AMA1 has been shown to generate a strong specific immune response and to provide effective protection against toxoplasmosis in mice [17]. Experimental studies revealed RON2 as a potential *Plasmodium* vaccine candidate [18]. Recent study has shown that a DNA vaccination that expresses RON4 and RON4 protein (RON4S2) induced immune responses, but failed to protect the mice against chronic toxoplasmosis [19]. The all above studies showed that AMA1, RON2 and RON4 might be potential vaccine antigens against toxoplasmosis.

Vaccine needs to include all the components capable of eliciting a protective immune response, notably the T- and B- cell epitopes. Attempts to develop a peptide vaccine that contains T- and B-cell epitopes for *T. gondii* have shown encouraging results [20,21]. Since bioinformatics has played a significant role in the analysis of protein epitopes, structures and functions, we used bioinformatics to predict the T- and B-cell epitopes of TgAMA1, TgRON2 and TgRON4 in this study.

The natural site of *T. gondii* infection is the mucosal surface of the intestine, and effective protection may require both mucosal and systemic immune responses. The intranasal route acts as an interesting alternative vaccination route and promoted both systemic and mucosal immune responses to an antigen [22-25], which can be used to target pathogens that invade far from the immunization site, such as the intestines [26].

Although host cell invasion has been well described at the ultrastructural and molecular level, the immunogenicity and protective efficacy of AMA1, RON2 and RON4 are still poorly understood. In this study, we predicted and synthesized the peptides of AMA1, RON2 and RON4 containing the T- and B-cell epitopes of *T. gondii*. We also comparatively evaluated the immune responses and protective efficacy of a single peptide of AMA1 (A1), RON2 (R2) or RON4 (R4), mixtures of two peptides (A1 + R2, A1 + R4 or R2 + R4) and a mixture of three peptides (A1 + R2 + R4) in BALB/c mice via intranasal immunization for the first time.

## Methods

### Epitopes prediction and peptides synthesis

The secondary structure and the B cell epitopes of AMA1, RON2 and RON4 of *Toxoplasma gondii* were predicted by the bioinformatics software of the DNAStar Protean system and the Bcepred online prediction tool [27,28].

Based on the results of these methods, we chose the peptides that have good hydrophilicity, satisfactory flexibility, high accessibility and strong antigenicity, but not the α-helix and β-folded sheets, which do not easily interact with antibodies and generally do not act as epitopes. We also predicted the potential B cell epitopes of TgAMA1, TgRON2 and TgRON4 by using the DNAMAN v6 software [29]. We used the online service SYFPEITHI (http://www.syfpeithi.de/bin/MHCServer.dll/EpitopePrediction.htm) to predict the Th cell epitopes of TgAMA1, TgRON2 and TgRON4. Based on the results obtained with these methods, the overlap regions of the predicted results were regarded as the potential T/B combined epitopes of TgAMA1, TgRON2 and TgRON4.

The chemically synthesized peptides listed in Table 1 were obtained from a peptide specialty laboratory (China Peptides Co., Ltd), which purified peptides by HPLC and verified their chemical identity by mass spectrometry. The concentrations of peptide solutions were determined via quantitative amino acid analysis using a Biochrom 20 Amino Acid Analyzer (Pharmacia Biotech). The purity of peptides was >98% in the lyophilized form.

**Table 1:** **The sequences of the peptides of T/B combined epitopes from** ***T. gondii*** **RH strain**

**Source of peptide** ^**#**^	**Amino acid position**	**Sequences**	**Numbers of amino acid**	**Molecular weight**
AMA1	41-55	CAELCDPSNKPGHLL	15	1596.84
RON2	119-148	LTAGGPLPHGSWSWSGTPPEVQTTGGSQIS	30	2993.26
RON4	343-374	KEQFFQFLQHLSADYPKQVQTVYEFLGWVADK	32	3891.43

^**#**^AMA1: Apical membrane antigen-1; RON2: Rhoptry neck protein 2; RON4: Rhoptry neck protein 4.

### *Toxoplasma gondii* strain

The tachyzoites of the virulent *T. gondii* RH strain were used to challenge mice, and were provided by the Peking University Health Science Center (Beijing, China). The parasites were maintained and collected from the peritoneal cavity of infected specific-pathogen-free (SPF) BALB/c mice as previously described [24,25].

### Mice and ethics statement

Female BALB/c mice were purchased from the Institute of Laboratory Animals, Chinese Academy of Medical Science (Beijing, China). All the mice were maintained under specific-pathogen-free conditions at the Centre of Laboratory Animals with a 12-hour light/dark cycle and provided with rodent feed and water *ad libitum*. The mice were acclimated for one week prior to the experiments. The animal protocols were approved by the Laboratory Animal Use and Care Committee of Shanxi Medical University (Permit No: SXMU-2011-16) and the Ethics Committee on Animal Research of the Shanxi Medical University (Protocol #: 20110320-1). All surgery was performed under sodium pentobarbital anaesthesia, and all possible efforts were made to minimise suffering of experiment mice.

### Immunization and challenge

A total of 176 female BALB/c mice aged 6 weeks were randomly divided into eight groups (22 mice per group). All mice were intranasally immunized with 30 μg AMA1, 30 μg RON2, 30 μg RON4, 15 μg AMA1 plus 15 μg RON2 (A1 + R2), 15 μg AMA1 plus 15 μg RON4 (A1 + R4), 15 μg RON2 plus 15 μg RON4 (R2 + R4) or a mixture of three peptides (10 μg of each peptide, A1 + R2 + R4). The peptides were suspended in 20 μl of sterile PBS, while control mice received 20 μl of sterile PBS alone. Each dose of immunogen was instilled into the nostrils of mice with a micropipettor (10 μl /nostril). All animals were vaccinated three times on days 0, 14 and 21.

Two weeks after the final inoculation (on day 35), 6 mice in each group were anesthetized with sodium pentobarbital (1.5%, 0.1 ml/20 g weight). Blood samples were collected from the mice in each group via retro-orbital plexus puncture, and the sera were stored at -70°C for further analysis. The spleens were collected under aseptic conditions to perform lymphocyte proliferation assays, and the culture supernatants were used for cytokine assays. Mucosal washes, including nasal, vesical and intestinal washes, were collected using PBS and stored at -70°C for secretory IgA (SIgA) assays.

Two weeks after the last immunization, another 10 mice from each group were challenged orally (with a feeding needle) with a lethal dose (4 × 10^4^ tachyzoites per mouse) of *T. gondii* RH strain. The symptoms and survival times of the challenged mice were monitored and recorded thrice daily for 30 days. To evaluate the effect of the immunization on the tissue tachyzoite burdens, the remaining 6 mice from each group were orally challenged with a nonlethal dose (1 × 10^4^ tachyzoites per mouse) of *T. gondii* strain RH. On the 30th day after being challenged, the mice were anesthetized with sodium pentobarbital, and the numbers of tachyzoite in the livers and brains were measured using a sensitive real-time quantitative PCR (qRTPCR) method as previously described [25,30].

For mice survival analysis, the infected mice were monitored at 8 am, 2 pm and 8 pm daily regarding their physical appearance, such as displaying rough coat, decreases in appetite, weakness/inability to obtain feed or water and depression. When these conditions were observed, a mouse would be moved to an isolated cage for further husbandry; if obvious suffering, such as struggling or whining was observed, the mouse would be sacrificed through ether inhalation. No obvious suffering was observed in this study.

### Sera antibodies determination

Antigen-specific IgA, IgG and IgG subclasses were analyzed by enzyme-linked immunosorbent assay (ELISA) as previously described [24]. Briefly, each well of a 96-well microtiter plates was coated overnight at 4°C with 100 μl peptide diluted in 0.05 M carbonate buffer (pH 9.6) at the optimal concentrations of 10 μg/ml for the AMA1, RON2 or RON4 groups, 12 μg/ml for the A1 + R2, A1 + R4 and R2 + R4 groups (6 μg/ml each peptide) or 15 μg/ml for the A1 + R2 + R4 and control groups (5 μg/ml each peptide). Murine sera diluted 1:300 in PBS was applied to the wells, and the plate was incubated at 37°C for 2 h, followed by the addition of goat anti-mouse IgG- or IgA-horseradish peroxidase conjugated antibody as a secondary antibody (Sigma), which was diluted 1:2000 or 1:1000. To analyze the subclasses, the sera were diluted 1:300, applied to the plates and developed with goat anti-mouse IgG1- or IgG2a-horseradish peroxidase conjugated antibody (Proteintech Group, Inc., USA) diluted at 1:2000. The results were expressed as OD_492_ using an ELISA plate reader and determined in duplicate for each serum sample from at least two independent ELISAs.

### Spleen cell proliferation assay

Spleen cell proliferation was assayed as previously described [25]. In brief, 5 × 10^5^ cells per well were cultured in triplicate in 96-well plates containing RPMI-1640 medium supplemented with penicillin-streptomycin (1 mM) and 10% fetal calf serum (FCS). The culture was stimulated with either 10 μg/ml of peptide, 5 μg/ml of concanavalin A (Con A) as a positive control or medium alone for proliferation. The plates were incubated in 5% CO_2_ at 37°C for 4 days. Next, 10 μl of CCK-8 reagent (Dojindo Laboratories, Japan) was added to each well, and the plate was incubated for 3 h. The optical density was then determined at 450 nm using an ELISA reader. The spleen cell proliferative responses were quantitated using a stimulation index (SI), which was calculated as the ratio of the average OD_450_ of the stimulated cells to the average OD_450_ of the unstimulated cells.

### Spleen cell cultures and cytokine assays

Splenocyte suspensions were prepared from six mice in each group 2 weeks after the last immunization, and 1.5 × 10^6^ cells/well were cultured in 24-well plates in triplicate in RPMI-1640 containing 10% FCS as described previously [24,25]. The culture was stimulated with 10 μg/ml peptide. The cell-free supernatants were harvested and assayed for interleukin-2 (IL-2) and IL-4 activities at 24 h, while the IL-10 activity was assessed at 72 h and the interferon-gamma (IFN-γ) activity was assessed at 96 h. The concentrations of IL-2, IL-4, IL-10 and IFN*-*γ were determined by a commercial ELISA kit (PeproTech, USA) according to the instructions provided by the manufacturer. The sensitivity limits of detection of IL-2, IL-4, IL-10 and IFN-γ were 16, 16, 47 and 23 pg/ml, respectively.

### Mucosal washes collection and SIgA determination

Prior to collecting the samples, the mice were deprived of food for 8 h to deplete the intestinal contents. Nasal washes were collected as described previously [25,31]. Briefly, the trachea was surgically exposed. A micropipette was then passed through the larynx and nasal cavity and a total of 0.6 ml of sterile PBS was flushed five times (0.1 ml per time) per mouse. The intestinal washes were collected using previously described protocols [24,25,32]. The small intestine sample was washed with 3.0 ml PBS. To collect the vesical washes, the bladder was exposed and rinsed gently with sterile PBS using the tip of a micropipette through the urethra. A total of 0.6 ml of sterile PBS was flushed gently in and out of the bladder, and all fluids were collected by micropipette. The bladder was flushed six times (0.1 ml per flush). Every 0.1 ml PBS was re-aspirated into the micropipette and re-injected into the bladder for a total of three cycles before final withdrawal.

All mucosal washes were centrifuged at 1000 × *g* for 10 min at 4°C to remove any tissue, fecal matter and cellular debris and were stored at -20°C until assayed. The titers of SIgA in the nasal, vesical and intestinal washes were also detected by ELISA at 492 nm as described above.

### Statistical analysis

The mean of each variable (IgA, total IgG, IgG1, IgG2a, IFN-γ, IL-2, IL-4 and IL-10) was compared between the different groups using one-way ANOVA. All statistical analyses were performed using the SPSS software for Windows version 13.0. The differences were considered statistically significant at *P* < 0.05. The tachyzoite burdens and survival times for vaccinated and control mice were compared using the Kaplan-Meier method. Levels of significance of the differences between groups were determined by the Student’s unpaired *t* test. Two-sided *P* values < 0.05 were considered to indicate statistical significance. Tests of normality for the data within each group were analyzed by the Shopiro-Vilk, *P* values were > 0.10.

## Results

### Sera antibodies responses

**Figure 1:**
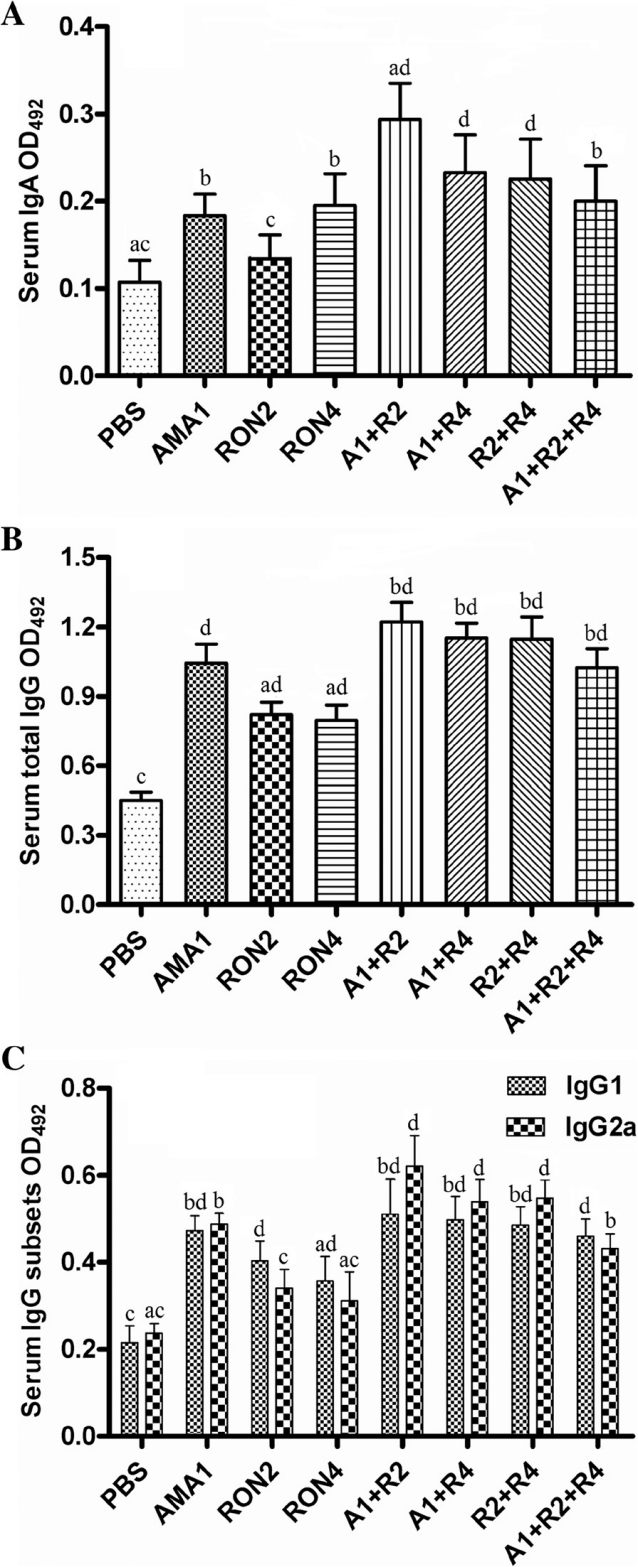
**Nasal immunization induces antigen-specific IgA and IgG responses in sera.** Serum samples were collected and diluted 1:300 to analyze the **(A)** IgA, **(B)** total IgG, **(C)** IgG1 and IgG2a contents by ELISA 14 days after the last immunization. The results are expressed as the means of the OD_492_ ± SD (n = 6) and from the two - time determinations. a vs. b, *P <* 0.05; c vs. d, *P* <0.01.

**Figure 2:**
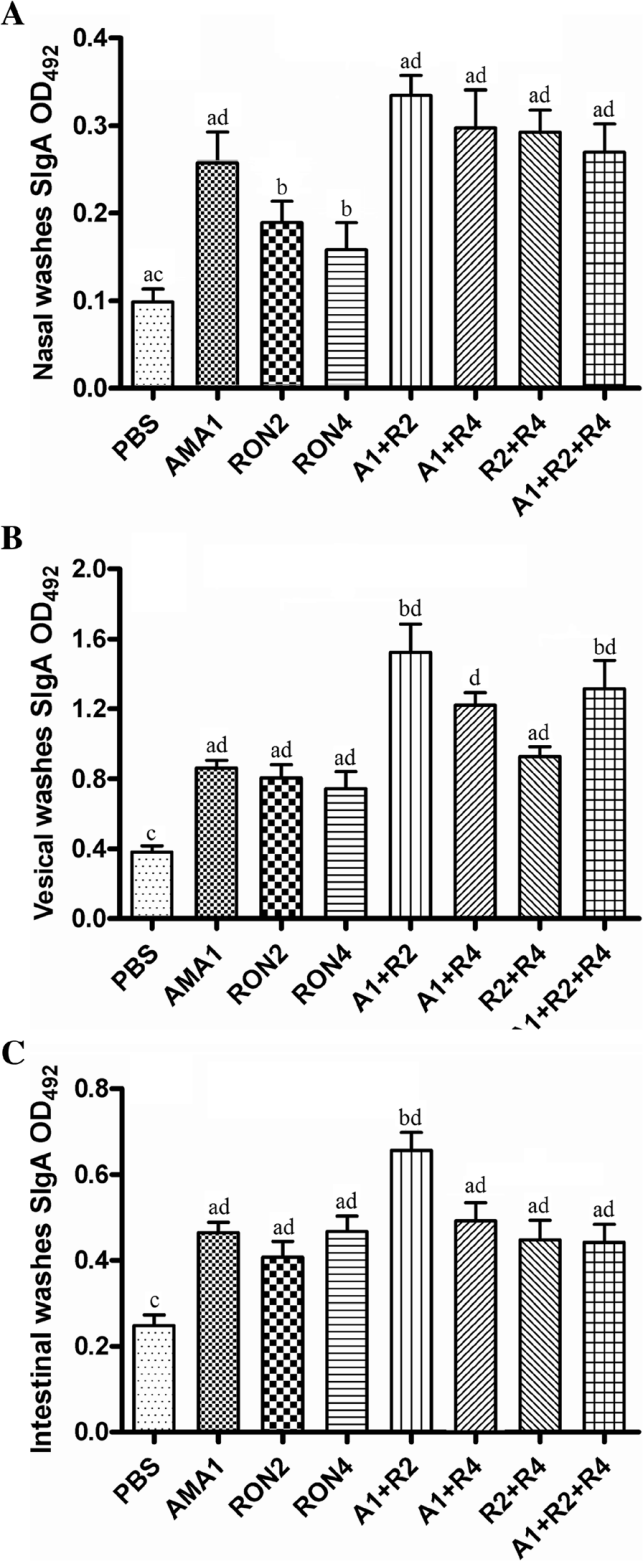
**Nasal immunization induces antigen-specific SIgA responses in mucosal washes.** Detection of SIgA in **(A)** nasal, **(B)** vesical and **(C)** intestinal washes from immunized mice by ELISA. Washes were obtained from six mice of each group 14 days after the last immunization. The results are expressed as the means of the OD_492_ value and standard deviation of the samples. a vs. b, *P <* 0.05; c vs. d, *P* <0.01.

Significantly high levels of IgA were detected in the sera from all immunized mice except the RON2 group when compared with the controls (*P* < 0.05 or *P* < 0.01). Among the immunized groups, the highest titer was generated in the A1 + R2 group, which was higher than the single peptide groups and A1 + R2 + R4 group (*P* < 0.05) (Figure 1A). All mice immunized with peptides developed higher total IgG levels, followed in order by the A1 + R2, A1 + R4 and R2 + R4 groups, in comparison to either control group, RON2 group or RON4 group (*P* < 0.05 or *P* < 0.01) (Figure 1B). The distributions of serum IgG1 and IgG2a are shown in Figure 2C. With the exception of the levels of IgG2a of mice from the RON2 and RON4 groups, the IgG1 and IgG2a levels of immunized mice were significantly higher than those of the control group (*P* < 0.05).

### Spleen cell proliferation responses *in vitro*

Spleen cells from 8 groups of mice were prepared 2 weeks after the last immunization to assess the proliferative responses to the peptides. As shown in Table 2, the splenocyte stimulation indices (SI) from the immunized groups were higher than that of the PBS group (*P* < 0.01). A1 + R2 immunization had the strongest activity vs all other groups. In addition, splenocytes from each experimental and control group proliferated well in response to the ConA (data not shown).

**Table 2:** **Lymphocyte proliferation and cytokine production by splenocytes stimulated with peptides**

**Groups** ^**#**^	**Lymphocyte SI** ^**##**^	**Cytokine production (pg/ml)** ^**##**^			
		**IFN-γ**	**IL-2**	**IL-4**	**IL-10**
PBS	0.76 ± 0.04 a	54.96 ± 12.07 a	57.17 ± 8.64 a	67.17 ± 6.43 a	84.13 ± 6.43
AMA1	2.04 ± 0.12 c	103.09 ± 18.99 b	156.58 ± 12.47 b	147.75 ± 11.67 b	102.25 ± 14.67
RON2	2.14 ± 0.24 c	97.39 ± 13.21 b	147.33 ± 11.92 b	136.42 ± 19.22 b	100.55 ± 19.22
RON4	2.21 ± 0.19 c	94.53 ± 16.60 b	134.42 ± 16.30 b	145.58 ± 12.98 b	97.75 ± 12.98
A1 + R2	3.17 ± 0.35 c	156.62 ± 17.25 c	219.71 ± 17.11 c	190.17 ± 21.11 c	110.13 ± 11.11
A1 + R4	2.21 ± 0.31 c	116.52 ± 16.60 b	183.84 ± 14.62 c	172.08 ± 16.22 b	111.51 ± 16.22
R2 + R4,	2.24 ± 0.40 c	123.36 ± 16.21 b	165.75 ± 24.20 b	174.83 ± 41.98 b	110.81 ± 11.98
A1 + R2 + R4	2.54 ± 0.32 c	108.52 ± 21.21 b	162.34 ± 17.11 b	174.17 ± 21.11 b	109.75 ± 11.11

^#^ *n* = 6 per group.

^##^Splenocytes from mice were harvested 2 weeks after the last immunization. SI represents stimulation index, calculated as the ratio of the OD_450_ of stimulated cells to the OD_450_ of unstimulated cells. The values for IFN-γ were obtained at 96 h, while the values for IL-2 and IL-4 were obtained at 24 h, and the values for IL-10 were obtained at 72 h. The results are presented as the mean ± SD of three replicate experiments. a vs b: *P <* 0.05; b vs c: *P <* 0.05; a vs c: *P <* 0.01.

### Cytokines production *in vitro*

The cell-mediated immunity produced in the immunized mice was evaluated by measuring the amount of cytokines (IFN-γ, IL-2, IL-4 and IL-10) in the supernatants of stimulated splenocyte cultures from mice of all groups. As shown in Table 2, significantly high levels of IFN-γ, IL-2 and IL-4 were detected in mice from all immunized groups compared with the control (*P* < 0.05). In addition, mice immunized with A1 + R2 had highest levels of IFN-γ, IL-2 and IL-4 than those of the single and mixture peptide groups (*P* < 0.05). However, the production of IL-10 did not statistically differ among all groups (*P* > 0.05).

### SIgA production in mucosal washes

The titers of SIgA in the nasal, vesical and intestinal washes of immunized mice were analyzed two weeks after the last immunization. Significantly higher titers of SIgA were detected in the mucosal washes from all immunized mice compared with the control (*P* < 0.05 or *P* < 0.01). As shown in Figure 2A, the mice immunized with peptides cocktail, especially A1 + R2, produced higher levels of SIgA in nasal washes (*P* < 0.05). Among the three single peptide groups, the highest titer was generated in the AMA1 group and the lowest was RON4 group.

The A1 + R2 immunized mice developed the highest levels of SIgA in the vesical washes among all groups. Moreover, the level of SIgA in the vesical washes of mice immunized with the three mixtures (A1 + R2 + R4) was higher than those of the single peptide groups and the R2 + R4 group (*P* < 0.05) (Figure 2B). As depicted in Figure 2C, the highest levels of SIgA were detected in the intestinal washes of mice immunized with A1 + R2, which were significantly higher than those of the other groups (*P* < 0.05 or *P* < 0.01).

### Protection against nonlethal and lethal *T. gondii* infection

**Figure 3:**
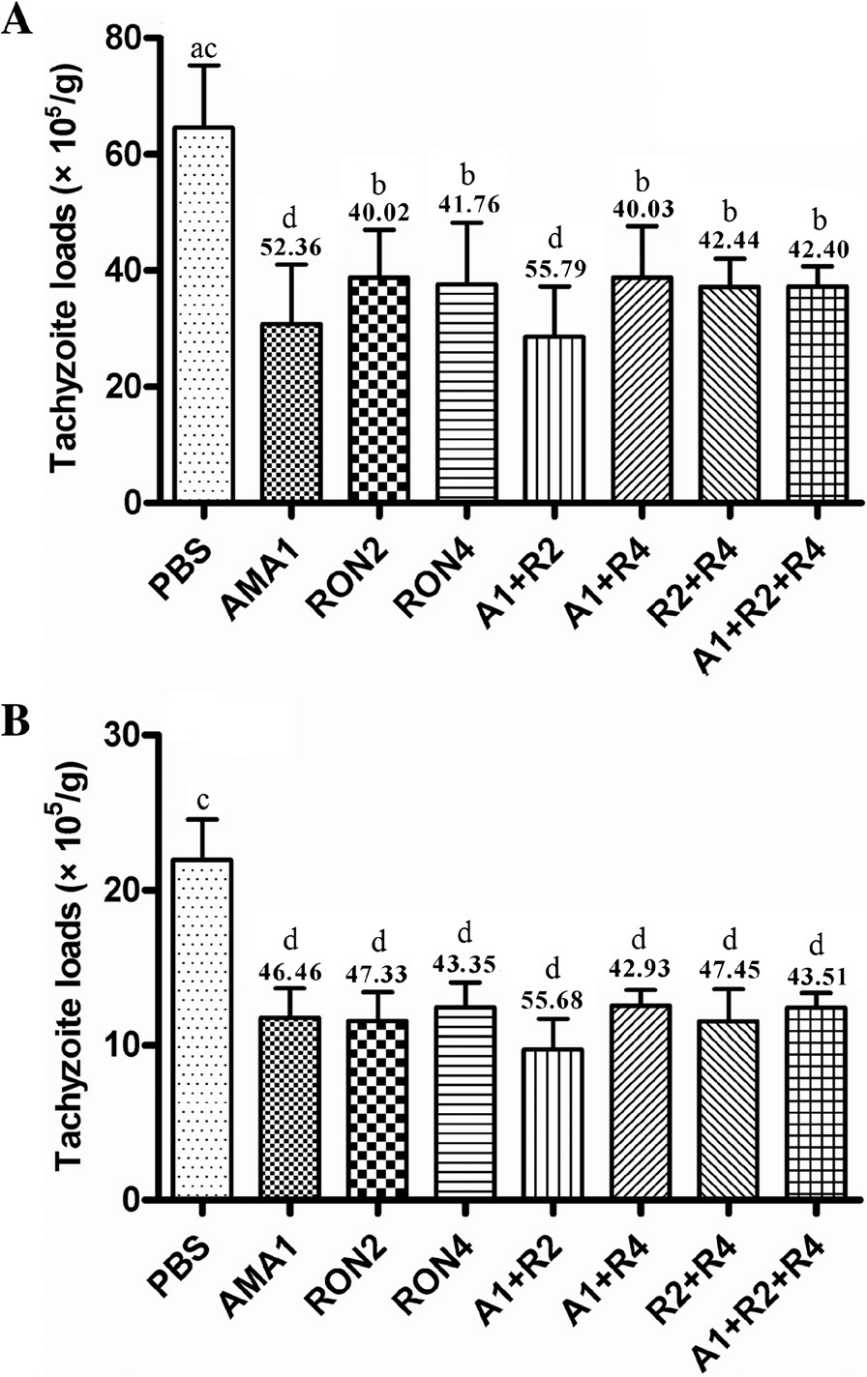
**Assessment of the reduction of tachyzoite loads in liver and brain. (A)** Liver and **(B)** brain tachyzoite burdens one month after challenge (mean ± SD, n = 6). The tachyzoite loads (×10^5^/per gram tissue) in the liver and brain of mice immunized after challenge with 1 × 10^4^ tachyzoites of *T. gondii* strain RH per mouse. The data columns in the illustration represent the reduction (%) of tachyzoite loads in the liver and brain of mice immunized compared with the values of the control group (PBS). The counting of parasites has been done twice for each tissue from per mouse. Values are means ± SD. a vs. b, *P <* 0.05; c vs. d, *P* <0.01.

To evaluate the efficacy of the peptide antigens against *T. gondii* infection, the numbers of tachyzoites in the livers and brains in the mice were determined one month post-peroral challenge. As shown in Figure 3, the tachyzoite loads in the liver and brain of all mice immunized with peptides were significantly lower than those in the PBS group (*P* < 0.05). Furthermore, the lowest tachyzoite loads in liver and brain were observed in the A1 + R2 group (*P* < 0.01); their reduction rates were 55.79% and 55.68%, respectively, when compared to the PBS group. Additionally, the reduction among the immunized groups did not statistically differ.

**Figure 4:**
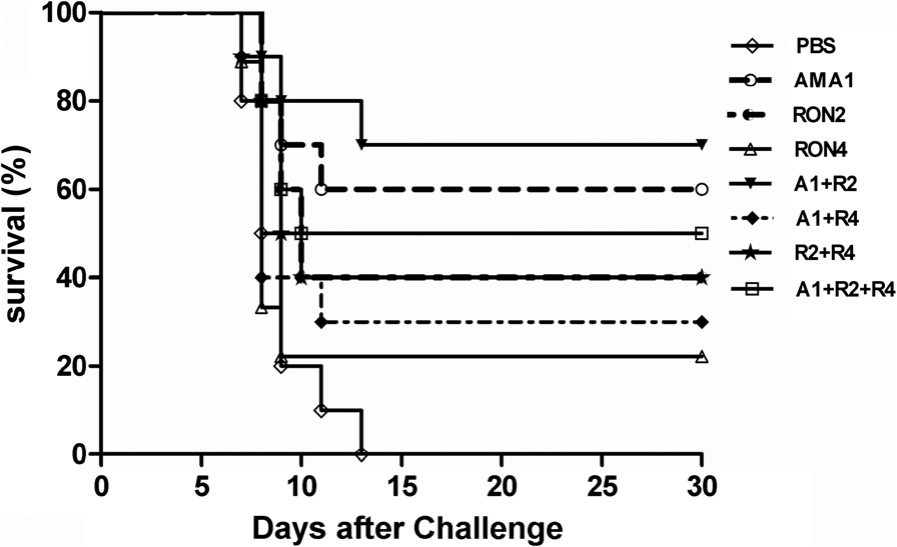
**Evaluation of survival curves against the lethal challenge.** Survival curves of immunized mice of the 8 groups (n = 10) were monitored daily after challenge with 4 × 10^4^ tachyzoites of *T. gondii* RH strain for 30 days post-challenge.

The survival rates of the mice were recorded daily following oral challenge for 30 days. The survival curves of all groups of mice are shown in Figure 4. Although the survival rate of all mice immunized with peptides were extended compared to mice from the control group, which died within 13 days, the survival rates of A1 + R2 (70%), AMA1 (60%), A1 + R2 + R4 (50%) and RON2 (40%) immunization reached significant level (*P* < 0.05).

## Discussion

Epitope-based vaccines should contain both T- and B-cell epitopes that will serve to induce proper immune responses [33]. In the present study, we focused on the AMA1, RON2 and RON4 involved in the invasion of *Toxoplasma gondii*. We predicted the potential T/B combined epitopes of TgAMA1, TgRON2 and TgRON4 by the bioinformatics method and evaluated the immune responses and protective efficacy of a single peptide or mixtures of peptides by contrast.

As a mucosal pathogen, the natural site of *T. gondii* infection is the mucosal surface where it can replicate efficiently [34]. By taking advantage of the common mucosal immune system (CMIS) [35], mucosal vaccination can elicit immune responses in multiple mucosal effector sites that are far from the immunization site [36]. Therefore, preventing infection at these sites via mucosal active vaccines is a promising and rational approach for vaccine development. Intranasal immunization is an effective and safe way of providing a disseminated mucosal immunity as well as systemic immunity [23,37].

An efficient mucosal vaccine should promote both mucosal and systemic immune responses [35,38]. The mucosal immune system can induce potent protective immunity against harmful pathogens to avoid mucosal colonization and pathogen invasion [39]. Secretory IgA (SIgA) is the main humoral mediator of the mucosal first-line defense system, and it is locally produced in the effector tissues of the mucosal immune system [40]. To evaluate mucosal immune responses, the levels of antigen-specific SIgA in the nasal, vesical and intestinal washes of immunized mice were examined in this study. We found a strong SIgA antibody response in the nasal and vesical washes of mice immunized with peptides. Moreover, the levels of SIgA in washes of mice immunized with mixtures of peptides, especially the A1 + R2 mixture, were significantly enhanced compared to the single peptide immunization. The intestinal IgA system is the best-understood contributor to mucosal immunity. In our study, the strongest SIgA antibody response in the intestinal washes was observed in mice immunized with A1 + R2. Taken together, the above results imply that a mixture of peptides, especially the mixture of two peptides (A1 + R2), was more effective in eliciting a mucosal immune response than a single peptide. Moreover, SIgA antibodies have been shown to reduce *T. gondii* infection of human enterocytes *in vitro*, which suggests that SIgA secretion is also important in defending against pathogen invasion into the intestinal mucosa [41,42].

System immunity plays a significant role in the defense against pathogens. Consistent with previous reports [42,43], our results showed that mice immunized with peptides had high specific IgG and IgA antibodies in sera. Moreover, the reactions of IgG and IgA of mice immunized with peptide mixtures were significantly enhanced compared to the single peptide immunization. Specific antibodies seem to be important in controlling *T. gondii* infection because the antibodies produced by B cells inhibit the attachment of the parasites to the host cell receptors, and macrophages kill intracellular Ab-coated tachyzoites [44,45]. Additionally, the splenocyte from the peptide immunized mice proliferated more rapidly than those of the control group, and the mice immunized with A1 + R2 showed the strongest increase.

Ideal vaccine candidates should induce both protective cellular Th1 and humoral Th2 responses [46]. The immune regulatory factors IFN-γ, IL-2 and IgG2a are associated with Th1-type responses, and IL-4, IL-10 and IgG1 are associated with Th2-type responses [47,48]. In this study, mice immunized with the peptides exhibited a mixed Th1/Th2 response with high titers of antigen-specific IgG1 and IgG2a antibodies. Our results showed that immunization with mixtures of A1 + R2 peptides can enhance the Th1 or Th2 mediated immunity, as evidenced by high levels of IFN-γ, IL-2 or IL-4 compared with the control group or single peptide groups. However, the production of IL-10 was not statistically different between different groups (*P* >0.05). Thus IFN-γ-mediated immune responses are necessary to control both the acute and chronic phases of *T. gondii* infection [49,50]. IL-4 may be involved in controlling *T. gondii* tachyzoites in the brain [51].

During the evaluation of protection potency, the highly virulent RH strain of *T. gondii* was used as the challenging infection in this study. All mice immunized with peptides showed significantly protective efficacy against nonlethal *T. gondii* infection compared with the control (*P* < 0.05). Furthermore, the mice immunized with A1 + R2, AMA1, A1 + R2 + R4 and RON2 against lethal *T. gondii* infection showed a significantly higher survival rate (*P* < 0.05); in particular the mice immunized with A1 + R2 showed the highest reduction of tachyzoite loads in the liver (55.79%) and the brain (55.68%). These results were consistent with previous reports that indicated that a DNA vaccine expressing RON4 and recombinant protein RON4 vaccine failed to protect the mice against oral challenge with *T. gondii* cysts [19].

A growing body of current research has focused on cocktail or multi-antigenic vaccines [18,52]. The enhanced immune responses and protective efficacy induced by mixtures of peptides in this study, especially A1 + R2, coincide with the results of the above-described studies, and the results also indicated that AMA1-RON2 interaction may play a key role in invasion by *Toxoplasma gondii*. This means that the combination of AMA1 and RON2 would be a better solution to fight against *T. gondii* induced immune reaction.

It is worth noting that the enhanced immune responses and protective efficacy induced by mixtures of two peptides, especially AMA1 and RON2 mixture, were more powerful and efficient than the mixture of three peptides. What are the reasons leading to this result? The analysis of the factors showed that the doses of mixture of three peptides group (10 μg each peptide) were less than doses of mixture of two peptides (15 μg each peptide) and it suggests that when antigen dose is too low, even if the three antigens are mixed, a better immune effect is not achieved.

## Conclusions

The intranasal immunization of mice with a single peptide (AMA1, RON2, RON4) or a mixture of peptides (A1 + R2, A1 + R4, R2 + R4 and A1 + R2 + R4) has been shown to induce effective mucosal and systemic immune responses and to elicit partial protection against acute and chronic *T. gondii* infection. Moreover, our study indicated that a mixture of peptides, especially the A1 + R2 mixture, was more powerful and efficient than a single peptide. These results demonstrated that AMA1, RON2 and RON4 containing T- and B-cell epitopes can be considered possible candidate antigens against toxoplasmosis, and a combination of peptides as a mucosal vaccine strategy is essential for future *Toxoplasma* vaccine development.
